# Data describing the cofactor additives effect on bioelectrocatalytic activity of «crude» extracts

**DOI:** 10.1016/j.dib.2020.105513

**Published:** 2020-04-22

**Authors:** M.V. Dmitrieva, E.V. Zolotukhina

**Affiliations:** a Institute of Problems of Chemical Physics, Chernogolovka, Russia; b Moscow Institute of Physics and Technology, Dolgoprudny, Russia

**Keywords:** biofuel cell, mediated bioelectrocatalysis, «crude» extracts, new type of bioelectrocatalyst, enzymes, glucose oxidation. E.Coli

## Abstract

«Crude» extracts obtained via simple ultrasonic disintegration of microbial cell membrane are perspective bioelectrocatalysts. This extract contains all the necessary enzymes and cofactors required for oxidative or reductive conversion. The technology of synthesis of «crude extract» is simpler and less costly in comparison with technology of obtaining pure enzymes. Dialysis of the obtained extracts was performed with different molecular weight cut-off (3.5 kDa, 12-14 kDa, 25 kDa, 50 kDa). The obtained data show that after dialysis extracts lose their dehydrogenase and bioelectrocatalytic activity due to the loss of cofactors. However, the addition of NAD and NADP cofactors leads to a recovery of activity. The obtained data demonstrate that the concentration of the cofactor directly affects the rate of the bioelectrocatalytic reaction. Also, the obtained data indicate that the composition of the enzyme systems of the extract includes succinate dehydrogenase. Analyzing this data set can provide insight on increase of the electrocatalytic activity of a new type of bioelectrocatalyst.

Specifications table

**Table:** 

Subject	Electrochemistry
Specific subject area	A new type of bioelectrocatalyst obtained via simple ultrasonic disintegration of microbial cell membrane
Type of data	Table Figure
How data were acquired	Data on dehydrogenase activity were obtained by the photocolorimetric method using the photometer Ephos-9305. Data on electrocatalytic activity were obtained by electrochemical measurements using the potentiostat Autolab PGSTAT 101.
Data format	Raw, analyzed
Parameters for data collection	Extracts from 6-hour cultures of *E.Coli* BB
Description of data collection	Dehydrogenase activity was determined by photocolorimetric method. Electrocatalytic activity was determined by electrochemical measurements in potentiostatic mode.
Data source location	Chernogolovka/Moscow region/Russia
Data accessibility	Data are included in this article

## Value of the data

• The data are important and innovative in the area of electrocatalysts for biofuel cells and biosensors. The obtained data relate to the improvement of electrochemical characteristics of a new type of bioelectrocatalysts developed by the authors in previous works.
• The data presented highlight the principal perspective of application of «crude» extracts as a new type of bioelectrocatalyst. Such data are attractive for researchers working on bioelectrocatalysis.
• Obtained data may help to understand processes occurring during bioelectrocatalytic oxidation of glucose. On the basis of this data can improve the electrocatalytic activity of a new type of bioelectrocatalyst.

## 1 Data Description

**Fig. 1:**
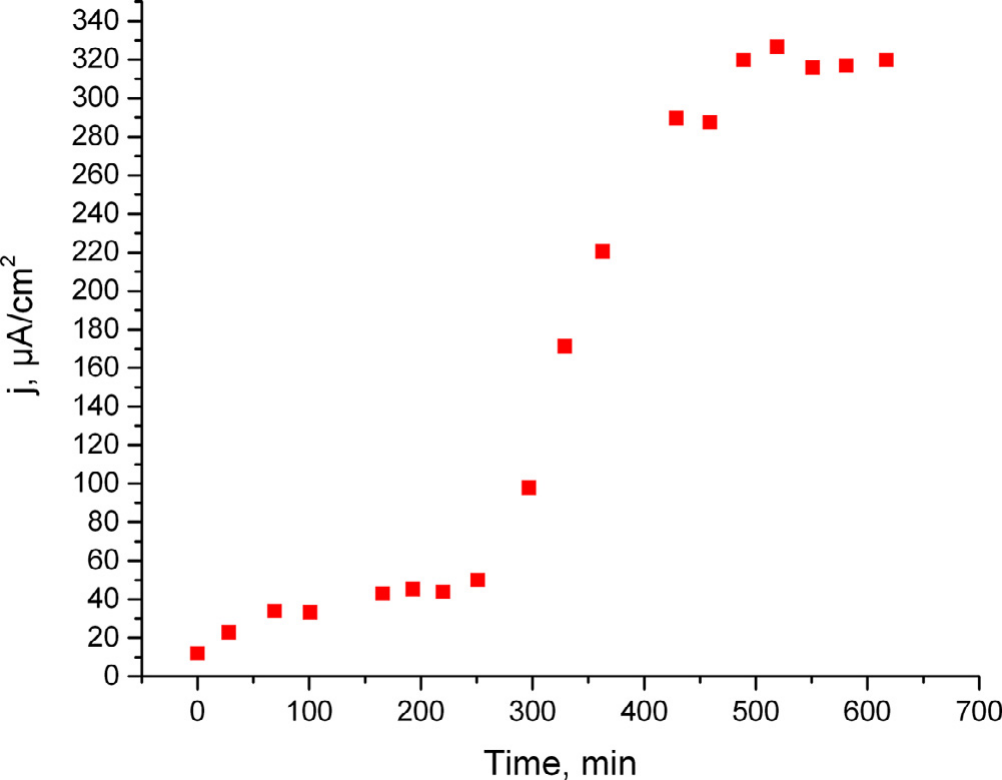
The time of the current output to the stationary mode. The measurements were carried out under optimal conditions for this reaction [2]. (see the raw data in the file Figure1)

**Fig. 2:**
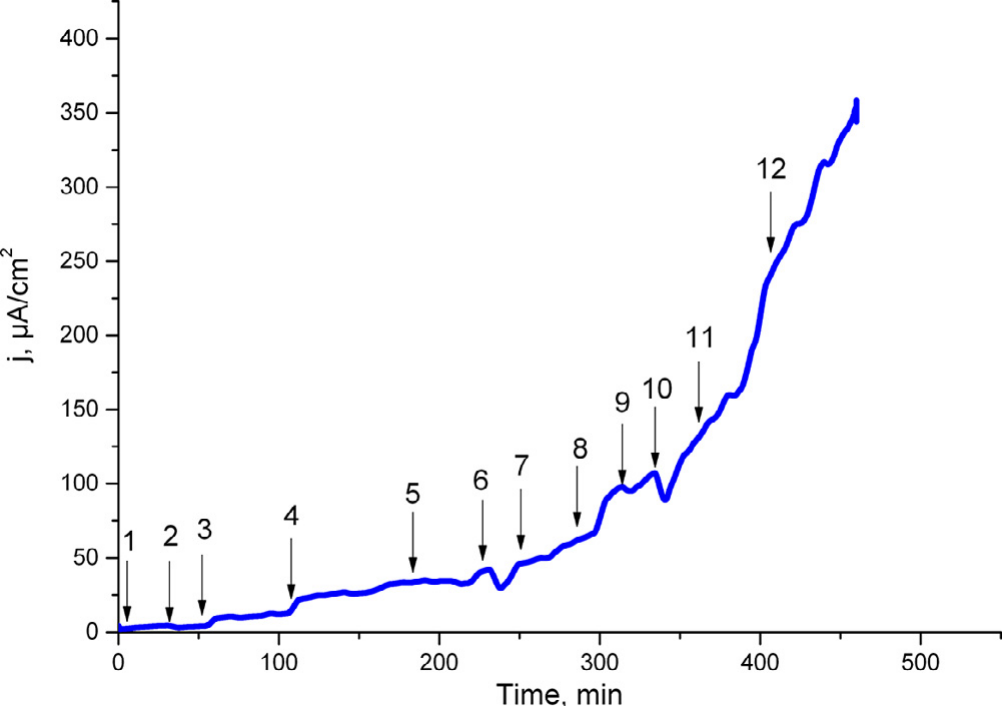
Electrocatalytic activity of extracts after dialysis with 3.5 kDa MWCO with addition of glucose and different amounts and type of cofactors. System: 0.5 М potassium phosphate buffer, рН 7.6, 0.6 ml of extract+4.6 mM glucose+5 mM K_3_Fe(CN)_6_+ Х М cofactor. ***1****-* without cofactor, ***2*** - 3.6*10^−7^ NAD, ***3*** - 2.05*10^−6^ NAD, ***4*** - 3.13*10^−6^ NAD, ***5*** - 3.5*10^−6^ NAD, ***6*** -6*10^−6^ NAD, **7** - 1*10^−5^ NAD, ***8*** - 2*10^−5^ NAD, ***9*** -3*10^−5^ NAD, **10** - 4*10^−5^ NAD, ***11*** - 6*10^−5^ NAD, ***12*** - 1*10^−4^ NAD (see the raw data in the file Figure2)

**Fig. 3:**
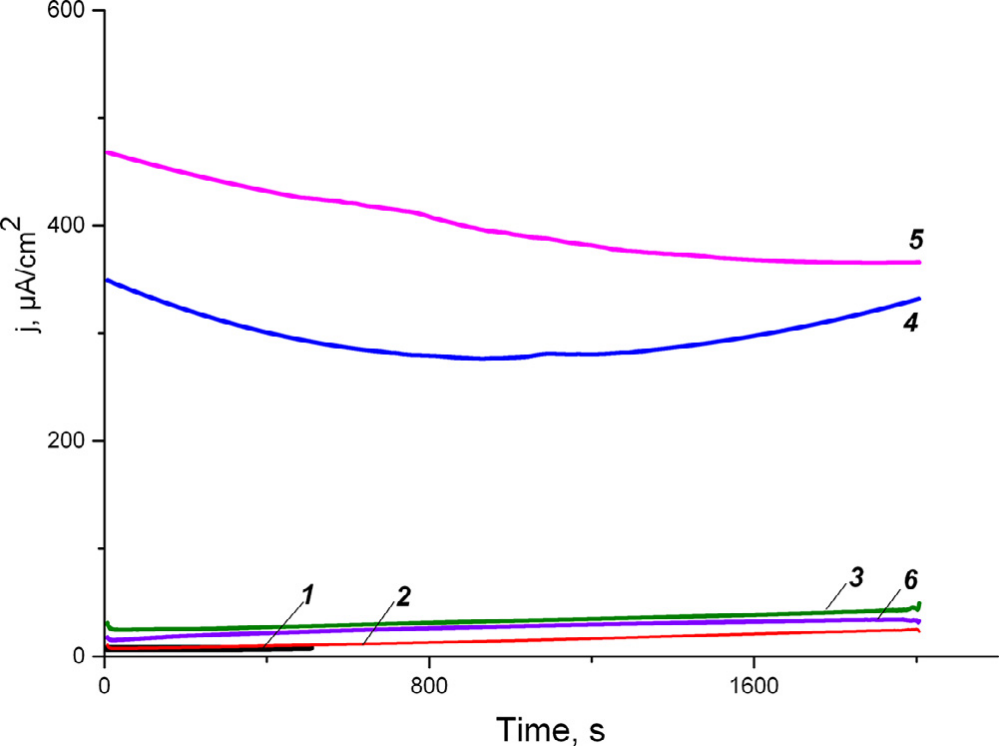
Electrocatalytic activity of extracts before and after dialysis with 3.5 kDa MWCO without addition of glucose. System: 0.5 М potassium phosphate buffer, рН 7.6, 0.6 ml of extract +5 mМ K_3_Fe(CN)_6_+Х М cofactor. *1* – after dialysis without cofactor (reaction 1), *2* – after dialysis + 1*10^−5^ M NAD, *3* - after dialysis + 1*10^−5^ M NAD+1*10^−5^ M NADP, *4* –after 15 hours from the incubation of the reaction *1, 5 –* after 3 days from the incubation of the reaction 1, *6* –before dialysis (initial extract). (see the raw data in the file Figure3)

We present data of dehydrogenase and bioelectrochemical activities of «crude» extract obtained via simple ultrasonic disintegration of microbial cell membrane [1, 2] after / without dialysis treatment and with / without cofactor additives. Such «crude» extract was prepared as described in our previous work [1]. First of all, we set the output time of the current resulting from the biocatalytic reaction of glucose oxidation to the stationary mode. As one can see it is about 8 hours (Fig. 1). We made the dialysis of such extracts with different MWCO (molecular weight cut-off). Then we measured the dehydrogenase activity of «crude» extracts before and after dialysis (Table 1). After dialysis (already at 3.5 kDa) there is a complete loss of dehydrogenase activity. However, after the addition of the NAD cofactor, the activity reappears in all 4 samples. These results correlate with data on electrocatalytic activity of extracts before and after dialysis due to extracts after dialysis and without cofactor addition have no electrochemical activity. A cofactor is a non-protein chemical compound or metallic ion that is required for an enzyme's activity as a catalyst [5]. So without cofactors enzymes cannot catalyze reactions effectively [3]. After dialysis the cofactors that are necessary for activity are washed out [4]. For the extract after dialysis with 3.5 kDa MWCO with the addition of glucose 4.6 mM and 1 * 10^−5^ M NAD current density was 49.5 µA /cm^2^ (Fig. 2), and without the addition of glucose for the same extract at the same concentration of cofactor-24.1 µA /cm^2^ (Fig. 3).

**Table 1:** Dehydrogenase activity of «crude» extracts before and after dialysis.

Sample	Dehydrogenase activity (mg of formazane/ml)
Extract without dialysis	0.02
Extract after dialysis with 3.5 kDa MWCO	0
Extract after dialysis with 50 kDa MWCO	0
Extract after dialysis with 3.5 kDa MWCO with addition of a cofactor NAD	0.02
Extract after dialysis with 50 kDa MWCO with addition of a cofactor NAD	0.02

This suggests that the concentration of the substrate affects the initial reaction rate. For the extract after dialysis, it is small because the glucose is partially washed off after dialysis.

**Fig. 4:**
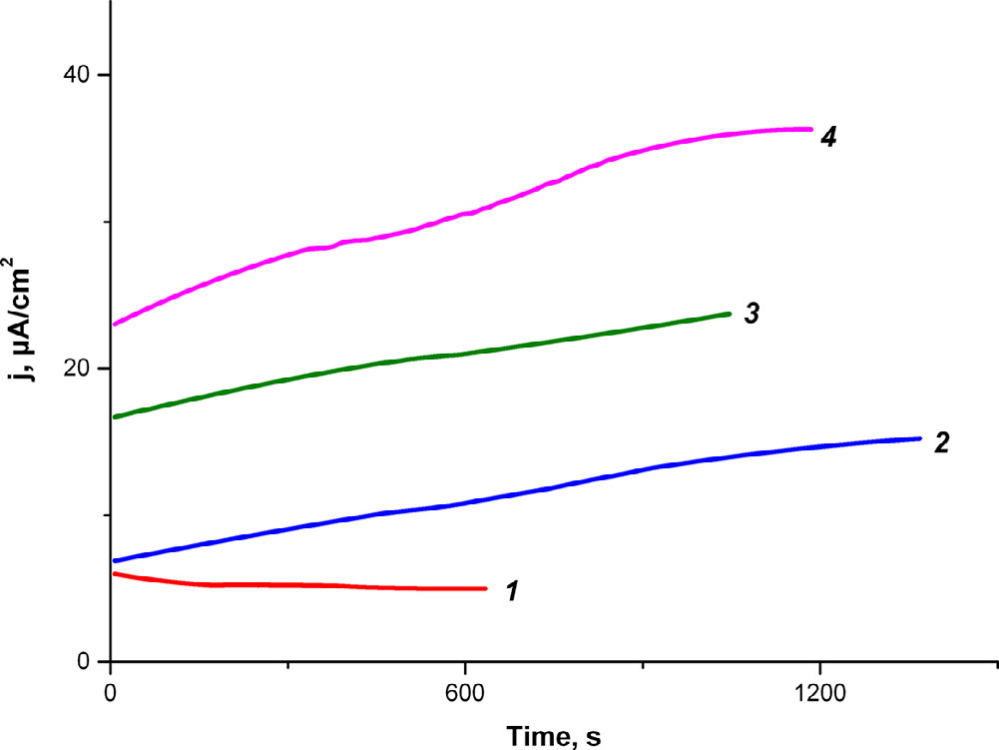
Electrocatalytic activity of extracts after dialysis with 50 kDa MWCO. System: 0.5 М potassium phosphate buffer, рН 7.6, 0.3 ml of extract+4.6 mM glucose+5 мМ K_3_Fe(CN)_6_ + Х М NAD+Х М NADP. ***1*** – extract+mediator+glucose, ***2*** – *1* +1*10^−5^ M NAD, ***3*** – *2*+2*10^−5^ M NAD, ***4*** – *3*+2*10^−5^ M NAD +1*10^−5^ M NADP. (see the raw data in the file Figure4)

For the extract after dialysis with 50 kDa MWCO without cofactor addition small current responses were also observed (Fig. 4). When the cofactor NAD was added at a concentration of 1*10^−5^ M, the specific current density increased to 230 µA/ cm^2^ * mg^−1^, which is comparable to the specific current density (287 µA/cm^2^*mg^−1^) for the extract after 3.5 kDa dialysis at the same cofactor concentration. When the cofactor NAD was added at a concentration of 2*10^−5^ M, the current density increased to 435 µA/cm^2^*mg^−1^, which is comparable to the current density (390 µA/cm^2^*mg^−1^) for the extract after dialysis of 3.5 kDa at the same cofactor concentration (Table 2).

**Table 2:** Summary table of current responses obtained in the bioelectrochemical system with the extracts after dialysis.

The concentration of the cofactor	j, µA/cm^2^	j specific, µA/cm^2^*mg^−1^
Extract after dialysis with 3.5 kDa MWCO		
1*10^−5^ NAD	50	287
2*10^−5^ NAD	68	391
3*10^−5^ NAD	97	559
4*10^−5^ NAD	112	648
6*10^−5^ NAD	160	924
Extract after dialysis with 50 kDa MWCO		
1*10^−5^ NAD	14	230
2*10^−5^ NAD	26	435

**Fig. 5:**
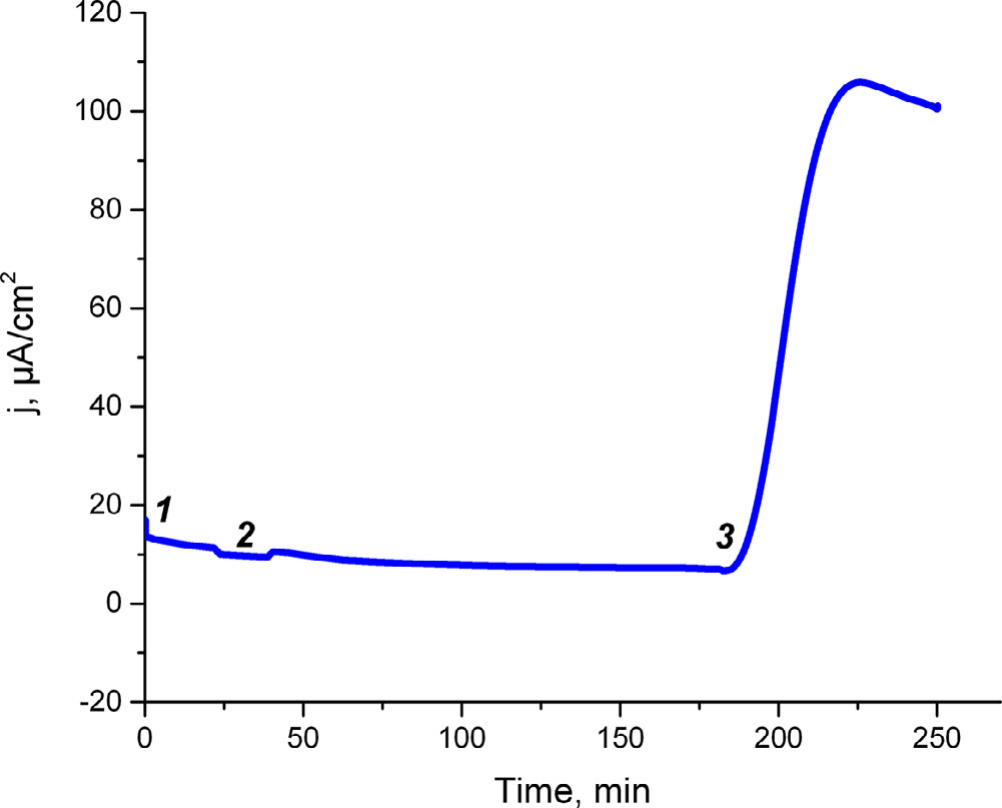
Electrocatalytic activity of extracts after dialysis with 3.5 kDa MWCO and addition of cofactor FAD. 1 – 5 mM K_3_Fe(CN)_6_+4.6 mM glucose+ 0.6 ml of extract after dialysis. 2 - 1+1*10^−4^ FAD. 3 - 2+4.6 mM succinic acid. (see the raw data in the file Figure5)

Cofactors NAD and NADP correspond to such enzymes as glucose 1-dehydrogenase EC 1.1.1.47 [6], glucose 1-dehydrogenase EC 1.1.1.118 [7]. Such enzymes are called NAD-dependent dehydrogenase [8]. But there are also FAD-dependent dehydrogenases [9]. So we measured the electrocatalytic activity of extracts after dialysis with 3.5 kDa MWCO with addition of cofactor FAD. There was no significant current response in the system «mediator+extract+glucose» (Fig. 5, 1). However the addition of the FAD cofactor did not lead to an increase in current responses (Fig. 5, 2). But the addition of succinic acid led to an immediate increase in current responses (Fig. 5, 3).

Succinate dehydrogenase catalyzes the FAD-dependent oxidation of succinate to fumarate [10].

## 2 Experimental Design, Materials, and Methods

### 2.1 «Crude» *E.coli* extract preparation

Bacteria culture were cultivated in LB medium containing 1% peptone from Bacto™ («BD», USA), 0.5 % yeast extract from Bacto™ («BD», USA), 1 % NaCl and 0.1 % C_6_H_12_O_6_. The colony of *Escherichia coli BB* which is grown up on the solid medium (LB, 2 % agar) was inoculated in 10 ml of LB for obtaining overnight bacterial culture. The overnight bacterial culture (1 ml) was brought in 100 ml of LB in 500-ml flasks and grown up at 37°C at intensive aeration. Through 6 hours after the beginning of cultivation cells were centrifuged at 1700 g for 30 min. Centrifuged samples were resuspended in buffer solution (50 mM KH_2_PO_4_, pH 7.2; 1 ml of buffer solution per 50 ml of the grown-up culture). Then cells were disrupted by ultrasonic disintegrator UZDN – 2T («NPP Ukrrospribor», Ukraine) at a frequency of 22 kHz during five series for 10 seconds. Between series samples were cooled. The rude enzyme extract was obtained by centrifugation for 15 min at 15000 g. The extracts were stored at a temperature -20°С.

### 2.2 Dehydrogenase activity measurements

The dehydrogenase activity was assessed with 2,3,5-triphenyltetrazolium chloride (**TTC**), which was reduced to colored TTC formazane under the effect of dehydrogenases. The substrates and electron sources for TTC reduction was glucose. The reaction mixture contained 10 mg/mL protein, 33.3 mM substrate, and 0.17 % TTC in PB. The reaction was carried out at 37 °С; 4.2 volumes of mixture of ethanol and acetic acid (19: 1) were added to stop the reaction. The denaturated protein was precipitated by centrifugation, and the supernatant was taken to measure the amount of TTC formazane. The staining intensity of the solutions was determined with an Ephos-9305 plate Photometer (MZ Sapfir, Russia) at 532 nm. The amount of formazane was calculated by a calibration curve obtained for standard purified TTC formazane.

### 2.3 Electrochemical measurements

Electrochemical measurements were carried out by the use of potentiostat Autolab PGSTAT 101 in a standard three electrode cell containing 15 ml of buffer solution as electrolyte and saturated silver chloride reference electrode, a glassy carbon working electrode (disk, 0.07 cm^2^) and a platinum foil counter electrode. Reference and counter electrodes were separated from work solution via glass frit. Experiments were performed mostly under argon atmosphere by means of Schlenk line. Active agents (mediator, glucose, extract, cofactor) were added directly into the cell which was under pressure of argon (20 mbar). All electrochemical measurements were performed in potentiostatic mode (potential was 0.5 V). Chronoamperometric measurements were carried out with magnetic stirrer (150 rpm).

## Appendix Supplementary materials

Image, application 1
